# From Agonist to Antagonist: Modulation of the Physiological Action of Angiotensins by Protein Conjugation—Hemodynamics and Behavior

**DOI:** 10.3389/fphar.2021.772217

**Published:** 2021-11-03

**Authors:** Tatyana S. Zamolodchikova, Svetlana M. Tolpygo, Alexander V. Kotov

**Affiliations:** Physiology of Motivation Laboratory, P. K. Anokhin Institute of Normal Physiology, Moscow, Russia

**Keywords:** drug design, angiotensins, peptide–protein conjugates, GPCR, ligand–receptor interactions, artificial ligand, hemodynamics, behavior

## Introduction

The modern period of medicinal and pharmacologic development is characterized by significant achievements in the area of peptide-based drugs. The concept of peptide regulation of organism biological functions detected feasibility for the development of new highly efficient regulatory peptide-based drugs that has resulted in heavy growth of pharmacologic studies in the area of peptide drugs, including the agonists of receptors associated with G proteins (GPCR) (Wacker et al., 2017; Grieco and Gomez-Monterrey, 2019).

The use of peptides as drugs is limited due to their short half-life, rapid degradation, and high clearance, but in some cases, for example, for Giapreza, containing angiotensin II (AngII), a short plasma half-life may be clinically beneficial (Khalique and Ferguson, 2019). In order to improve pharmacologic properties of the peptide drugs, a special strategy of peptide conjugation with high molecular weight compounds, including proteins, is developed (Bumbaca et al., 2019; Davenport et al., 2020). A similar approach is used to create conjugate peptide vaccines for stabilization, delivery to the immune competent cells, and stimulation of the immune reaction; the target peptide antigen is conjugated with any protein, such as keyhole limpet hemocyanin or bovine serum albumin (BSA). An analogous method for vaccine creation is proposed for the prospective method of hypertension immune treatment: peptide–protein antihypertensive vaccines contain different factors of the renin–angiotensin system (RAS), including protein-conjugated angiotensins (Downham et al., 2003; Garay-Gutiérrez et al., 2021).

In pursuit of the goal of correcting pathologic conditions using synthetic peptide–protein preparations, it should be borne in mind that the incorporation of a peptide into an artificial macromolecular construct is inevitably accompanied with changes in its structural conformation characteristics due to new peptide–protein interactions. For this reason, the peptide hormone integrated into the peptide–protein complex is an altered ligand with globally modified signaling properties. There are cases when the use of GPCR-targeted peptide drugs leads to unexpected reactions and dangerous side effects (Allen and Roth, 2011).

Based on literature and own data, we show by the example of angiotensins–multifunctional factors of the RAS that the hormones included into the synthetic peptide–protein complexes could globally change their properties till reverse agonism and cause unpredictable physiological effects.

## Opinion

### Ligand–Receptor Interactions—Induced Conformity

It is known that ligand interaction with the receptor is implemented based on its structural conformity (Costa-Neto et al., 2016). Moreover, the molecular features and properties of the ligand have effect on the conformation state of the receptor, that in turn determines the function of many receptors, particularly, GPCR (Horiuchi et al., 2012). There are multiple conformations of the angiotensin receptor AT1R, some of which bind the receptor with signaling pathways, whereas others control its phosphorylation or internalization (Holloway et al., 2002). If a receptor is involved in several possible signal transduction pathways, the implementation of one pathway or another will be conditioned by the stabilization of the receptor conformation that meets the structural requirements or signatures of the given ligand (Chipens, 1980; Franco et al., 2021). The phenomenon of ligand-specific conformational states of the receptor has been called the functional selectivity or biased agonism (Costa-Neto et al., 2016). GPCR agonists, which mainly activate the β-arrestin pathway of signal transduction, are β-arrestin–biased agonists; moreover, G-protein–biased agonists mainly activating G-protein–dependent pathways of signal transduction are known (Saulière et al., 2012; Wingler et al., 2020). The concept of biased agonism has changed well-established notions about GPCR as one-dimensional regulators of the linear signal cascades and allowed considering them as multidimensional transducers, which can involve different signal paths and adjust them variously.

Obviously, the signatures of the native peptide and the same peptide included in the synthetic protein–peptide complex can be radically different. Therefore, during interactions with the conjugated macromolecular ligand, the conformation conditions of the receptor will significantly differ from the conditions induced by the appropriate natural ligand that can have an effect on functional selectivity (biased agonism). In comparison with native ligand–receptor interaction, the binding of a bulky peptide–protein complex to the receptor will have features conditioned by the spatial molecular landscape of the area of their contact on the cell surface. All abovementioned factors complicate forecasting biological effects caused by synthetic macromolecular ligands.

### Macromolecular AngII-Containing Ligands

In Torres-Tirado et al. (2013), the inverse ratio was established between the rate of AT1R internalization and desensitization from molecular mass of polymeric AngII-containing ligands. Based on the assumption that the high molecular weight ligand will be less susceptible to internalization, other authors (Pang et al., 2019) studied the signaling properties of the chemically synthesized AngII-BSA peptide–protein complex, in which 10–12 molecules of AngII are bound to one BSA molecule. Such a ligand was developed as less capable of internalizing agonist supposedly inhibiting the ß-arrestin–biased pathway for signal transduction, which, as is known, is activated with the involvement of internalized AT1 (Reiter et al., 2017). In comparison with native AngII, AngII-BSA significantly stimulated saline appetite, which is controlled by the activation of β-arrestin path (Daniels et al., 2009). Contrary to the initial assumptions, AngII-BSA turned out to be a biased agonist precisely in relation to the β-arrestin signal transduction pathway. Thus, the signaling properties of BSA-conjugated AngII differ significantly from the properties of native AngII; therefore, signal transduction pathways initiated by the AngII-BSA complex, as well as the corresponding reactions at the cellular and organismal level, can be largely unpredictable.

### Effects of Rat Immunization With the AngII-BSA Conjugate

When using the synthetic peptide drugs, the achievement of therapeutic effects mainly supposes their long-term use. Long-term and regular administration of signal peptides conjugated with proteins into an organism can result in unexpected or even negative effects. The properties of biologically active peptides in a complex with protein molecules are modified; moreover, such a macromolecular structure turns out to be highly resistant to destructive action of the enzymes and can be accumulated in the organism (Bumbaca et al., 2019). These considerations appear to be especially relevant in relation to use of peptide–protein drugs as vaccines developed for the correction of hypertension, where factors of the RAS, particularly AngII (Garay-Gutiérrez et al., 2021), are used as an antigenic determinant. In our studies (Tolpygo et al., 1990), it was shown that long-term (13 months) rat immunization with the AngII-BSA conjugate regardless of the titer of AngII-specific antibodies has long-term (up to 10 months) effects in relation to drinking behavior, pain sensitivity, and motor activity of animals. Regular injection of AngII-BSA contrary to native AngII resulted in the occurrence of significant dipsogenic effects (intensification of water consumption up to 74% from the initial level); increased pain threshold up to 67%, and reduced motor activity of the rats up to 30% from the initial level. In this experiment, no significant long-term effect on blood pressure was observed with either native AngII or AngII-BSA.

### Modulation of Angiotensin Activity by Conjugation With Proteins

Significantly, sometimes cardinally differing spectra of the activity of free and protein-bound angiotensins were demonstrated in a number of our works (Kotov et al., 2003; Pevtsova et al., 2008; Pevtsova et al., 2009; Tolpygo et al., 2012). For the study, central effector RAS – octapeptide AngII and its derivatives heptapeptide Ang (1–7) and hexapeptide AngIV were selected. Conjugates of these peptides with BSA (67 kDa) and protein S100b (20–28 kDa) in mole peptide/protein ratio 10–12/1 (BSA) and 4–5/1 (S100b) were synthesized. Influence of AngII, Ang (1–7), AngIV, and their conjugates with BSA and S100b on hemodynamic parameters (SBP and HR) and drinking behavior of the rats including complicated instrumental behavior are shown in Table 1.

**TABLE 1:** Effect of angiotensins and their conjugates with BSA and S100b on hemodynamics and behavior.

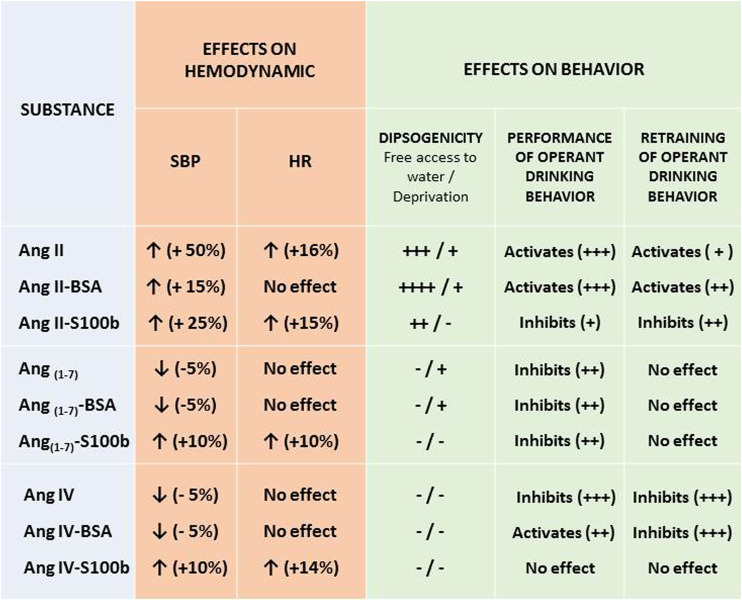

Notes: Hemodynamic effects: an increase (↑) or a decrease (↓) values are indicated, the percentage change in the measured parameters relative to the control is indicated in parentheses; behavioral effects: minus/plus means the absence/presence of an effect, the severity of which increases from minimum (+) to maximum (++++). The data were taken from Kotov et al., 2003; Pevtsova et al., 2008; Pevtsova et al., 2009; Tolpygo et al., 2012.

Compared to natural AngII, conjugated ligands markedly reduce the hypertensive effect and differently affect the heart rate. Dipsogenicity of the AngII-BSA significantly exceeds the dipsogenicity of AngII, whereas AngII-S100b is only a partial agonist in this respect. AngII’s property to activate the instrumental behavior of rats persists in AngII-BSA, while AngII-S100b has inhibited the appearance of instrumental skills.

Ang (1–7) has specific Mas receptors and has anti-hypertensive properties being a functional antagonist AngII (Haulica et al., 2005). This explains research interest to Ang (1–7) as to the object for the development of drugs for the treatment of coronary vascular diseases. The results of our studies have shown that the hemodynamic properties of Ang (1–7) bound to the protein can be reversed: Ang (1–7)-S100b conjugate has expressed hypertensive effects and accelerates the heart rate. Dipsogenic effects of Ang (1–7) are manifested in different ways in the composition of different macromolecular complexes.

Conjugation with a carrier protein can globally, up to functional inversion, change the properties of AngIV (Pevtsova et al., 2009) which has its own receptor, insulin-regulated aminopeptidase, and plays an important role in learning and memory mechanisms (Wright and Harding, 2019). Ang IV has cardioprotective effect, whereupon it is considered to be pharmacologically perspective peptide (Hussain and Awan, 2017). The effects of AngIV-BSA on hemodynamic parameters are similar to influence of native AngIV, which is weakly anti-hypertensive and does not influence the heart rate. In contrast, AngIV conjugated to S100b exhibited inverse agonist properties, significantly increasing blood pressure and increasing heart rate. In this experimental model, natural AngIV had an inhibitory effect on the instrumental behavior of animals, whereas the effects of AngIV complexes with proteins were significantly different from those of the free peptide (Table 1).

## Discussion

The design of new biomolecules, including protein–peptide conjugates for pharmacological objectives is widely used at the present time. Thus, clinical development of peptide–protein drugs–receptor agonists and drugs for directional delivery of drug preparations into tissues and organs or the creation of innovative vaccines for immune correction of some pathologic conditions supposes high molecular structure designing, where the target peptide is conjugated with the carrier protein (Bumbaca et al., 2019; Davenport et al., 2020). Peptide incorporation into macromolecular structures results in its structural modification due to conformation changes and new intramolecular interactions that inevitably affects its signaling properties.

The observed wide range of changes in the activity of angiotensins, included in peptide-protein complexes indicates the global influence of the protein component on signaling properties of the peptide hormones. For example, AngII–BSA significantly enhances drinking behavior, so it is logical to assume a potentiating effect of this conjugate, similar to native AngII, on hemodynamic parameters, such as blood pressure and heart rate (Kaschina and Unger, 2003). However, the observed effects of AngII-BSA on hemodynamic parameters are much weaker when compared with AngII. At the same time, this conjugate is even more dipsogenic than the native peptide (Table 1). This phenomenon can be due to functional selectivity or biased agonism. Actually, the receptor can generally interact with the whole spectrum of signal pathways, but ligands with affine property to this receptor can influence only some of them. Signal pathways controlling dipsogenic effects can incompletely coincide with the pathways of signal transduction, ensuring hypertensive reaction to AngII (Daniels et al., 2005). Receptor conformational states induced by the signature of the AngII-containing peptide–protein complex can differ significantly from the native states corresponding to free AngII, which will inevitably affect the functional selectivity and, ultimately, the ratio of the severity of the dipsogenic and hypertensive properties of the artificial macromolecular ligand. The nature of the protein component can dramatically change the properties of the protein-conjugated peptide also in relation to instrumental behavior, which can be, especially, clearly observed in the case of Ang II and Ang IV (Table 1).

The hemodynamic and behavioral effects of modified angiotensins described in this article, obviously, do not exhaust the full completeness of the body’s possible reactions to their use. Probably, in this respect, we observed only the tip of the iceberg. Since most regulatory peptides are multifunctional, as in the case of angiotensins, whose role is universal and concerns the functioning of all systems at all levels from cellular to behavioral (Haulica et al., 2005; Zamolodchikova et al., 2016; Crowley and Rudemiller, 2017), the use of artificial ligands containing such peptides as drugs can cause unpredictable reactions of various vital systems and organs. One would assume that this problem can be solved by focusing on the development of non-peptide analogs of endogenous peptides. However, as shown by the example of morphine and other opiates (Badal et al., 2018), the interaction of non-peptide ligands with opiate receptors leads to an imbalance in the processes of signal transduction and receptor desensitization as a result of their structural rearrangement induced by such ligands. The considerations presented here should be taken into account when achieving the goal of correcting pathologic conditions using drugs containing modified receptor ligands.
